# The abundance and persistence of *Caprinae* populations

**DOI:** 10.1038/s41598-022-17963-w

**Published:** 2022-08-15

**Authors:** Grant M. Harris, Matthew J. Butler, David R. Stewart, James W. Cain

**Affiliations:** 1 United States Fish and Wildlife Service, Albuquerque, NM USA; 2 US Geological Survey New Mexico Cooperative Fish and Wildlife Research Unit, Las Cruces, NM USA

**Keywords:** Population dynamics, Conservation biology

## Abstract

Stable or growing populations may go extinct when their sizes cannot withstand large swings in temporal variation and stochastic forces. Hence, the minimum abundance threshold defining when populations can persist without human intervention forms a key conservation parameter. We identify this threshold for many populations of *Caprinae*, typically threatened species lacking demographic data. Doing so helps triage conservation and management actions for threatened or harvested populations. Methodologically, we used population projection matrices and simulations, with starting abundance, recruitment, and adult female survival predicting future abundance, growth rate (λ), and population trend. We incorporated mean demographic rates representative of *Caprinae* populations and corresponding variances from desert bighorn sheep (*Ovis canadensis nelsoni*), as a proxy for *Caprinae* sharing similar life histories. We found a population’s minimum abundance resulting in ≤ 0.01 chance of quasi-extinction (*QE*; population ≤ 5 adult females) in 10 years and ≤ 0.10 *QE* in 30 years as 50 adult females, or 70 were translocation (removals) pursued. Discovering the threshold required 3 demographic parameters. We show, however, that monitoring populations’ relationships to this threshold requires only abundance and recruitment data. This applied approach avoids the logistical and cost hurdles in measuring female survival, making assays of population persistence more practical.

## Introduction

Worldwide, *Caprinae* populations receive much attention given their coveted trophy hunts and iconic symbolism for wild and remote places, yet many *Caprinae* populations are at risk of extinction^1^. For biologists to protect and restore *Caprinae* populations threatened with extinction, and manage populations not threatened, they often apply logistically difficult and sometimes controversial techniques such as harvest, predator control, and translocation. Appropriate execution of these techniques requires foundational information describing a *Caprinae* population’s chance of extinction, demographic weaknesses, and minimum abundance requirements.

Populations with demographic rates producing stable to growing populations may decline to extinction when their sizes cannot withstand large demographic swings from temporal variation and stochastic forces^2^. Therefore, understanding the minimum population size at which a population can persist without (or minimal) human intervention forms a key conservation parameter. For *Caprinae*, the minimum abundance target for adult females enables biologists to triage conservation actions across populations^3^. Conservation resources can focus on rebuilding threatened populations below the threshold, and non-threatened populations may be hunted or serve as sources for translocation. Relatedly, this metric assists threat evaluations, as populations above the minimum threshold are more resilient to local extinction events^4^.

We identify this minimum abundance threshold describing population persistence for *Caprinae*. Methodologically, we perform a population viability assessment (PVA; e.g.^5,6^), by developing a population projection matrix model to simulate population responses (population size and growth (λ) through time). We use matrix population models, as they form a conventional tool for predicting the growth of animal populations based on demographic parameters such as births, deaths, and age-structure^7,8^. The matrix approach is also commonly applied for identifying and assessing conservation strategies for endangered species^9,10^.

Although the 32 extant, autochthonous species of *Caprinae* have diverse behaviors, body sizes and inhabit different ecosystems, all females birth one offspring per year, by their second to third year (until death; twinning common in some species)^1^. We built our population viability model for *Caprinae* off these defining characteristics.

The urgency of conservation actions for most *Caprinae* populations far outpaces the rate of data collection^1^. Hence, the majority of *Caprinae* lack demographic information required to estimate population viability (e.g. mean and variability estimates of adult female survivorship and recruitment). We overcome the data deficiencies by simulating results using multiple combinations of starting abundances (varying from 10 to 250 adult females), mean recruitment (spanning 0–0.90) and adult female survivorship (0.50–0.98). Our simulations incorporated biological (temporal and environmental) variation and stochasticity, thereby quantifying uncertainties in future predictions. Estimating parameter variability relies on long-term datasets^11^, also nonexistent for most *Caprinae*^1^. Therefore, we quantified variation in recruitment and survivorship using data from prior field surveys and peer-reviewed literature on desert bighorn sheep (*Ovis Canadensis nelsoni*), as a proxy for other *Caprinae*.

The parameter values and combinations we employ correspond to empirical values reported from many different *Caprinae* populations. Indeed, estimating the minimum viable population (MVP) by evaluating a spectrum of biologically plausible values covers the differences in *Caprinae* social systems and environments influencing a given populations survival and recruitment values. We never assume that values for all *Caprinae* are similar, nor do we consider data from desert bighorn sheep representative for all *Caprinae*. We included model code and all simulation output, so any user can select demographic values of choice and examine the resulting changes in population persistence, or tailor an investigation to a specific population of interest.

Despite the copious literature defining minimum population sizes and building demographic simulations for a variety of species, prior studies rarely consider the practicality that managers require to inform on-the-ground work^12^. Such answers hinge on using data that managers can logistically acquire, affordably. Herein we implement this applied and practical approach.

We quantify and report the relationships between recruitment and adult female survival with population growth (λ), and therefore, the combinations of recruitment and survivorship required to attain a given λ, as recruitment and survival have most influence on *Caprinae* abundance^13,14^. Recruitment represents the number of offspring produced by reproductive females, each year, entering the yearling age class. Acquiring data describing recruitment is inexpensive and logistically simple. In contrast, survival data are usually derived from catching, marking (i.e., telemetry collars) and following adult females. The work is logistically challenging and expensive, making data describing survivorship scarce. It is also unlikely that biologists will acquire survivorship data for many *Caprinae* anytime soon. Therefore, while our simulations incorporate survivorship data, monitoring for the threshold abundance does not. Instead, we simplify results so biologists interested in determining when a population is secure from localized extinction can rely solely on accurate measures of abundance, abundance trend and recruitment.

Our results identify which demographic parameter generates the greatest impacts for increasing growth rate (λ) to recover an ailing population. We also show effects of translocation (adult female removals) on population abundance and trend of the source population, to identify populations sizes at which translocation is not detrimental. Lastly, we assess if survival or recruitment are useful for evaluating the efficacy of management actions, as the variability inherent to these parameters challenges such assessments.

For *Caprinae* managers, our results likely represent the sole information describing population projections and minimum abundance thresholds for the endangered *Caprinae* populations under their charge. For other wildlife and conservation practitioners, we exemplify a procedure for modeling population trajectories and identifying demographic thresholds for species with limited data.

## Results

### Demographic parameters

Wildlife agencies often produce lamb:adult female ratios (i.e. recruitment) from surveys. To quantify variability in recruitment, we analyzed recruitment data of desert bighorn sheep from three state agencies (USA): the Arizona Game and Fish Department (AZGFD), the California Department of Fish and Wildlife (CAFW), and the New Mexico Department of Game and Fish (NMDGF). The yearlings of *Caprinae*, of either sex, can be challenging to identify as their size and horn characteristics often mimic adult females. Agencies handle this situation differently. The CAFW and NMDGF report the number of yearling females identified but omit yearling males and unclassified yearlings. The AZGFD reports the number of male, female and unclassified yearlings. The NMDGF includes yearling females in the denominator of the lamb:ewe ratio but CAFW and AZGFD do not. Across the three states, the lamb:ewe ratios that omit yearlings from the denominator (L:E) and those that include yearlings in the denominator (L:EY) are generally similar (Table 1).

**Table 1:** Recruitment data from desert bighorn sheep populations during years with adult female (ewe) abundance ≥ 20. The ratios labeled L:E represent the mean lamb:ewe ratio with yearling counts excluded from the denominator, and L:EY represents the mean lamb:ewe ratio with yearling counts included in the denominator (90% CIs; LCL—Lower, UCL—Upper, SD indicates standard deviation). The column “*n*” reports the number of site-years included in the calculation. Acronyms are as follows: *AZGFD* Arizona Game and Fish Department, *CAFW* California Department of Fish and Wildlife, *NMDGF* New Mexico Department of Game and Fish.

Ratio	Agency	*n*	Mean	SD	LCL	UCL
L:E	NMDGF	35	0.35	0.15	0.30	0.40
L:E	CAFW	107	0.34	0.23	0.30	0.38
L:E	AZGFD	615	0.33	0.17	0.32	0.34
L:EY	NMDGF	35	0.33	0.15	0.28	0.38
L:EY	CAFW	107	0.33	0.22	0.29	0.37
L:EY	AZGFD	615	0.29	0.16	0.28	0.30

When yearlings counts are removed from the denominator of the L:E ratio, an unknown, incomplete and inconsistent fraction of yearlings are actually removed from each survey. For example, 46% of surveys for calculating L:E ratios from NMDGF (35 site/years) and 75% from CAFW (107 site/years) have ≤ 1 female yearling reported. In Arizona, 48% of surveys have ≤ 1 female yearling counted, 47% have ≤ 1 male yearling counted, 85% have ≤ 1 unclassified yearling counted, and 21% have ≤ 1 yearling counted in all three yearling categories (max of 3 total yearlings; 615 site/years). Across the 3 states, 38% of surveys recording ≥ 50 ewes do not report any female yearlings, and 70% report ≤ 5 female yearlings. The simplest (by avoiding misclassification issues) and most comprehensive (contains all yearling animals observed) correction is including yearlings in the recruitment ratio (lamb:(ewe + yearling); L:EY).

We estimated mean recruitment (L:EY) as 0.294 with a biological variance of 0.028 (*n* = 757; CV = 0.57). The mean and variance using the traditional L:E ratio (i.e., assumed exclusion of all yearlings from the denominator) was 0.33 and 0.033, respectively. Our subsequent analyses always used the biological variance with yearlings included in the ratio denominator.

The mean recruitment we employ aligns with values reported from other populations of *Caprinae,* and the variability in recruitment matches the reduced versions of variability we tested. For instance, populations of Dall’s sheep (*O. dalli dalli*) have lamb:(ewe + yearling) ratios ~ 0.30 with CVs exceeding 0.4^15^. Mountain goats (*Oreamnos americanus*) have mean kid:(yearling + nanny) ratios of 0.33 and CV = 0.21 (calculated from Table 1 found in^16^). Punjab urial (*Ovis vignei punjabiensis*) can have mean lamb:ewe ratios of 0.44 with CV = 0.18^17^, and Astor markhor (*Capra falconeri falconeri*) have mean kid:(yearling + nanny) ratios of 0.32 and CV = 0.30 (number of females ≥ 20; N = 8^18^). The variability in our recruitment data is likely higher than reported elsewhere for many other *Caprinae* populations, because our method accounts for inter-annual variability in the calculation (not calculating the mean variance).

We estimated the mean survival for adult female desert bighorn sheep as 0.82, with biological variance = 0.0127 (CV = 0.14; 31 site/years). This mean and variance in survivorship also corresponds with other *Caprinae* populations, such as Soay sheep (*Ovis aries*; mean = 0.87; CV = 0.15 [winter period]), mouflon (*Ovis gmelini*; mean = 0.84; CV = 0.13), and Dall’s sheep (mean = 0.88; CV = 0.11)^13,19–21^.

### Simulations and minimum abundance of adult females

**Figure 1:**
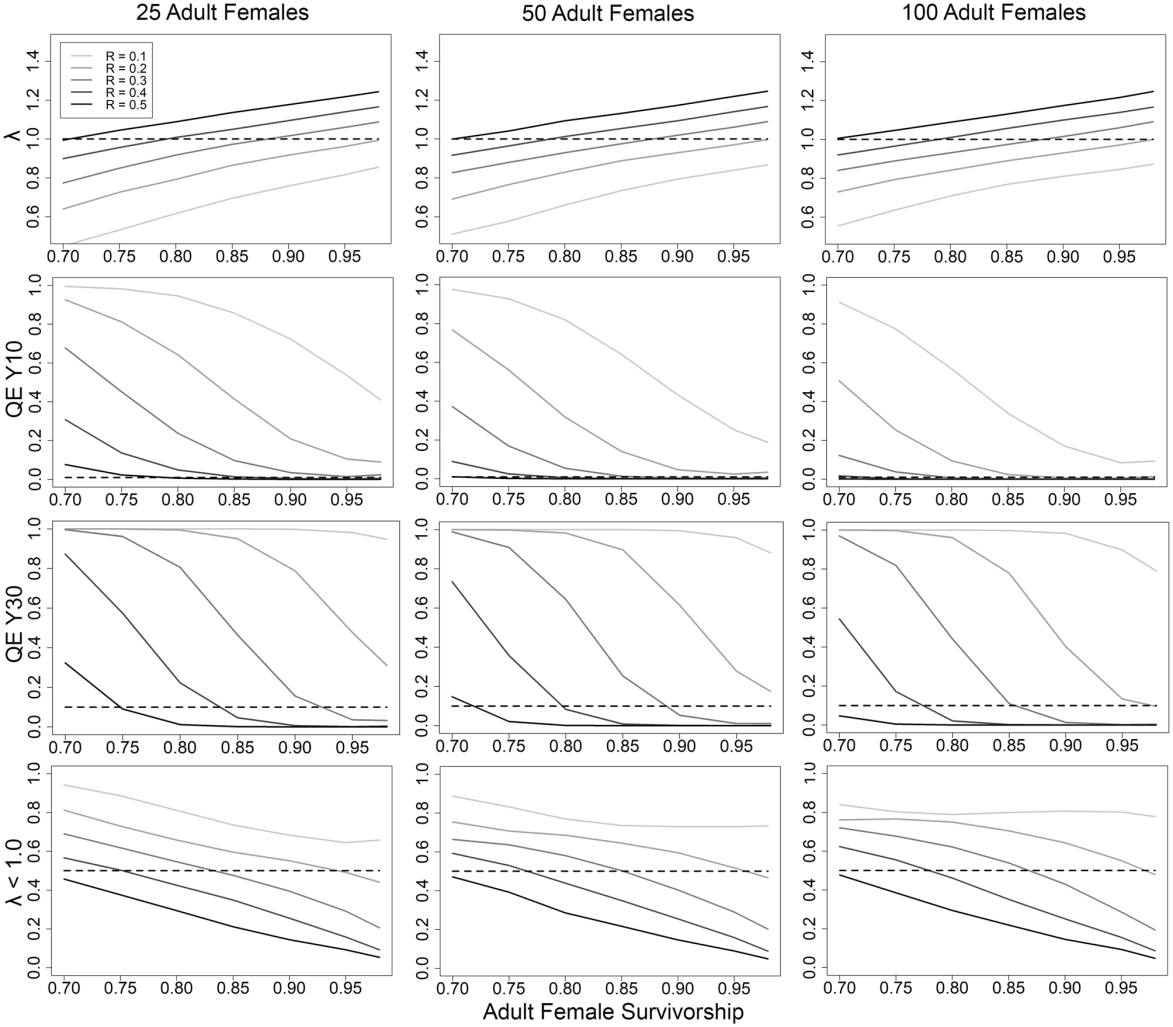
Relationships between recruitment (R), survival of adult females, starting adult female abundance, and population growth (λ; top row), the probability of quasi-extinction (*QE*; adult female abundance ≤ 5) at years 10 and 30 (middle rows) and the proportion of simulations resulting in mean λ < 1.0, based on 10,000 stochastic simulations of population trajectories. Dashed lines for *QE*(10) and *QE*(30) indicate the persistence threshold: 0.01 and 0.10 respectively.

We quantified the probabilities of λ < 1 and quasi-extinction at year 10 and 30 (*QE*(10); *QE*(30)), with different combinations of mean adult female survival, mean recruitment, and starting abundance of adult females, with the variance values fixed (Fig. 1). To achieve a stable population (λ ~ 1.0), the lowest recruitment can become is 0.20, provided an adult female survival of 0.98 (deterministic λ = 1.01; Table 2; Fig. 1). Alternatively, recruitment could be as high as 0.45 with a corresponding survivorship of 0.75 (deterministic λ = 1.01; Table 2; Fig. 1).

**Table 2:** Values of population abundance for adult females (N), recruitment (R), and survivorship of adult females (S) used in the 30-year population modeling simulations (10,000 simulations per R and S combination) to acquire mean population growth (λ) ~ 1.0, with lower and upper 90% CI’s (LCL, UCL), deterministic λ (λD), extinction probability at year 10 (E), quasi-extinction probability (*QE*; the probability that the population declines to ≤ 5 adult females at years 10 and 30), and the proportion of scenarios resulting in λ < 1.

N	R	S	λLCL	λ	λUCL	λD	λ < 1	E(10)	*QE*(10)	*QE*(30)
10	0.2	0.98	0.50	0.98	1.33	1.01	0.41	0.10	0.31	0.56
10	0.25	0.95	0.55	1.00	1.38	1.02	0.37	0.06	0.25	0.41
10	0.3	0.9	0.55	1.00	1.39	1.03	0.40	0.07	0.25	0.43
10	0.35	0.85	0.50	0.99	1.43	1.02	0.44	0.08	0.28	0.46
10	0.4	0.8	0.50	0.99	1.43	1.02	0.45	0.10	0.31	0.53
10	0.45	0.75	0.47	0.97	1.44	1.00	0.48	0.13	0.34	0.57
25	0.2	0.98	0.69	0.99	1.29	1.01	0.44	0.03	0.09	0.31
25	0.25	0.95	0.69	1.02	1.31	1.03	0.38	0.01	0.04	0.17
25	0.3	0.9	0.68	1.02	1.33	1.03	0.39	0.00	0.03	0.16
25	0.35	0.85	0.67	1.01	1.33	1.02	0.42	0.00	0.04	0.18
25	0.4	0.8	0.65	1.01	1.35	1.02	0.43	0.01	0.05	0.22
25	0.45	0.75	0.62	1.00	1.38	1.01	0.44	0.01	0.07	0.28
50	0.2	0.98	0.76	1.00	1.26	1.01	0.47	0.01	0.03	0.18
50	0.25	0.95	0.71	1.02	1.29	1.03	0.40	0.00	0.01	0.06
50	0.3	0.9	0.70	1.02	1.30	1.03	0.40	0.00	0.00	0.05
50	0.35	0.85	0.70	1.01	1.32	1.02	0.43	0.00	0.00	0.06
50	0.4	0.8	0.69	1.01	1.32	1.02	0.44	0.00	0.01	0.08
50	0.45	0.75	0.67	1.01	1.33	1.01	0.45	0.00	0.01	0.11
100	0.2	0.98	0.78	1.00	1.25	1.01	0.48	0.00	0.01	0.10
100	0.25	0.95	0.72	1.02	1.27	1.03	0.41	0.00	0.00	0.02
100	0.3	0.9	0.72	1.01	1.28	1.03	0.43	0.00	0.00	0.01
100	0.35	0.85	0.72	1.02	1.29	1.02	0.44	0.00	0.00	0.01
100	0.4	0.8	0.71	1.01	1.30	1.02	0.46	0.00	0.00	0.02
100	0.45	0.75	0.70	1.01	1.31	1.01	0.47	0.00	0.00	0.03

The variability surrounding λ is high, with confidence levels often spanning ± 30% (Table 2). The likelihood of *QE* helps represent this variability. Acquiring *QE*(10) ≤ 0.01 with survivorship ≥ 0.85, requires a population of 25 adult females with recruitment ≥ 0.4, a population with 50 adult females with recruitment ≥ 0.3, and 100 adult females with recruitment approximately ≥ 0.2 (Fig. 1). A *QE*(30) ≤ 0.10 occurs with survivorship ≥ 0.80 and recruitment ≥ 0.4 with 50 adult females, and survivorship ≥ 0.85 and recruitment ≥ 0.3 with 100 adult females (Fig. 1).

We also examined variability by quantifying the proportion of scenario iterations resulting in λ < 1 (Fig. 1, Table 2). Starting abundance has relatively small effects on this probability (Fig. 1). Attaining a ≤ 25% chance of population decline (stochastic λ) requires combinations of recruitment and survivorship such as survivorship ≥ 0.95 and recruitment 0.30, survivorship ≥ 0.90 and recruitment 0.40, or survivorship ≥ 0.85 and recruitment 0.50 (Fig. 1). Attaining a ≤ 10% chance of population decline requires survivorship ≥ 0.95 and recruitment ≥ 0.50 (Fig. 1).

We determined the minimum abundance of adult females required for ensuring population persistence, when the population has demographic rates consistent with a stable to slightly growing population (stochastic λ ~ 1; Supplementary Data S1). Results focused on *QE*, because starting abundance strongly affects this probability. With 25 adult females, populations have *QE*(10) ranging 0.03–0.09 and *QE*(30) 0.16–0.31 (Table 2). A population of 50 adult females must have intermediate levels of recruitment (0.25–0.40) and survivorship (0.80–0.95) for *QE*(10) ≤ 0.01 and *QE*(30) ≤ 0.10 (Table 2). With a starting abundance of 100 adult females, a population has *QE*(10) < 0.01 and *QE*(30) ≤ 0.10 for all simulations. For all starting abundances, the risk of *QE* increases with high recruitment and low survival (and vice-versa; Table 2). Based on these results, a population of 50 adult females forms a reasonable target to ensure population persistence.

**Figure 2:**
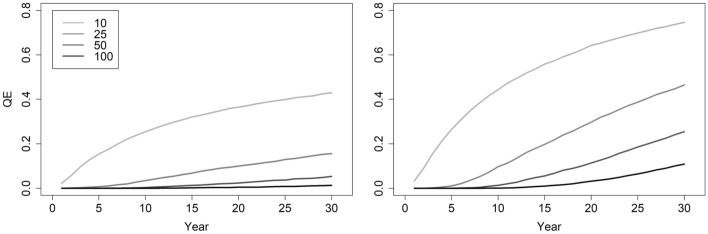
Plots portraying the probability of quasi-extinction (*QE*; mean abundance ≤ 5 adult females) of adult females based on 10,000 stochastic simulations of population trajectories. The left panel locks recruitment to 0.30 and survivorship at 0.90 (stochastic λ ~ 1.0) and the right panel has recruitment and survivorship at 0.30 and 0.85 respectively (stochastic λ ~ 0.97). Inset values indicate the starting abundances of adult females.

*QE* increases when populations exhibit modest decline, as when survivorship is 0.85 and recruitment 0.30, generating λ ~ 0.97. With λ in slight decline, the *QE*(10) risk for a population of 50 adult females remains nearly identical to simulations with λ ~ 1.0, yet *QE*(30) rises ~ 5 times (Tables 2, 3; Figs. 1, 2). A population of 100 adult females with λ ~ 0.97 has *QE*(30) 10 times larger than when λ ~ 1.00 (Tables 2, 3; Figs. 1, 2). With λ ~ 0.97, populations having a starting abundance of 60 or 70 adult females decline to 50 by year 8 and 16 respectively. Therefore, a population of 50 adult females remains a minimum threshold target, provided that frequent and accurate monitoring of abundance catches any decline.

**Table 3:** Likelihood of population extinction (E; year 10), and quasi-extinction (*QE;* mean abundance ≤ 5 adult females) at year 10 and 30 from 10,000 simulations for a population in slight decline (recruitment = 0.30; survivorship = 0.85; with λ ~ 0.97).

Abundance	LCLλ	Meanλ	UCLλ	E(10)	*QE*(10)	*QE*(30)
10	0.40	0.94	1.40	0.13	0.44	0.74
25	0.60	0.97	1.33	0.01	0.10	0.46
50	0.66	0.97	1.28	0.00	0.01	0.25
100	0.68	0.97	1.26	0.00	0.00	0.11
250	0.70	0.98	1.25	0.00	0.00	0.09

**Figure 3:**
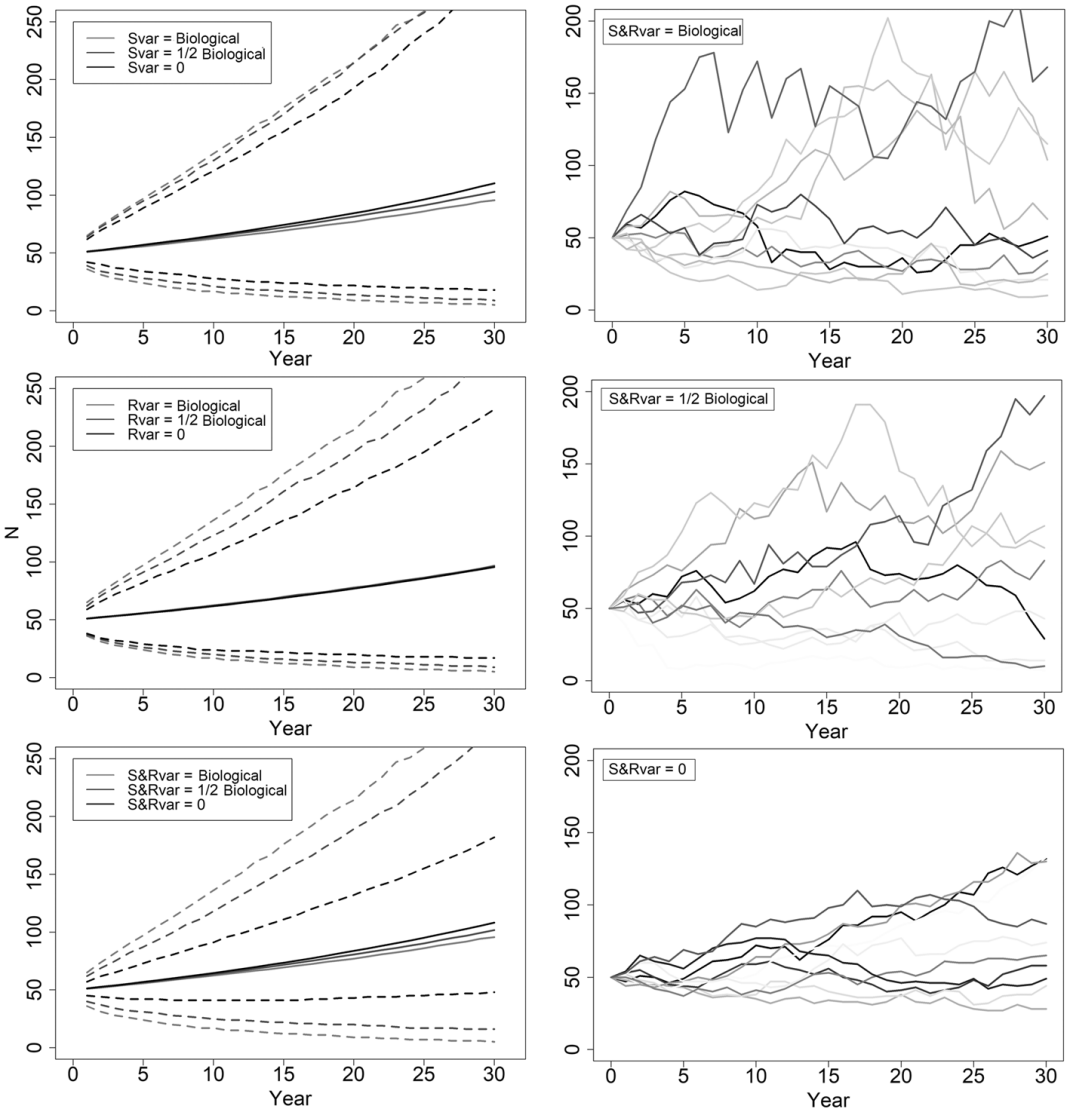
The effects of variability in adult female survivorship and recruitment parameters on the abundance estimates for adult females. Mean abundances are solid lines with dashed lines representing 90% confidence intervals. Upper left: Recruitment variance held at the biological value (0.028), and survivorship variance takes the biological value (0.0127), halved or eliminated. Middle left: Survivorship variance held at biological value, and recruitment variance takes the biological value, halved or eliminated. Bottom left: Variability of survivorship and recruitment vary from both biological, to half biological and variability in both removed. Right 3 panels: 10 randomized abundance simulations with the variability in survivorship (Svar) and recruitment (Rvar) biological, both half biological, and both removed (0). All scenarios have mean recruitment of 0.30 and mean survivorship of 0.90 (stochastic λ = 1.02).

Biological variation influences the potential trajectories of population growth or decline. Higher variability means more alternative paths, so reductions in variability typically provide greater assurances in a population’s projection. We found that arbitrarily reducing biological variation by half for recruitment or adult female survival barely changed the resulting variability surrounding λ (Table 4). Nor does this reduction in variability substantially influence a population’s trajectory (Fig. 3). Keeping biological variance for one parameter and eliminating variance for the other, or halving biological variation for both parameters, reduces variability surrounding a stable λ to 20%, with little effect on population trajectories (Table 4; Fig. 3). Reducing variability by 90% or eliminating it for both parameters generated confidence intervals spanning ~ 10% around a stable λ (Table 4; Fig. 3). Without biological variance, a population of 50 adult females could decline to 40 or increase to 90 adult females by year 10 (Fig. 3). Increases in biological variation raise *QE*, but effects do not manifest at year 10 (*QE*(10) always 0), and *QE*(30) changes are minimal (Table 4).

**Table 4:** Effects of biological variance (var; the component of variance due to temporal and environmental variation) in recruitment (R) and adult female survival (S) on the variability in λ. Results from 10,000 simulations with starting abundance (N) of 50, (year 10 of the 30 year simulation presented, with the values of extinction and quasi-extinction in year 10 essentially 0 (except *QE*(30), the quasi-extinction value at year 30)). Mean stochastic lambda (λ) with 90% CIs (LCL, UCL) and probability of scenarios resulting in stochastic λ < 1 (deterministic λ = 1.03). Biological variation was derived from the empirical recruitment and survival data.

N	R	S	Rvar	Svar	λLCL	λMean	λUCL	λ < 1	*QE*(30)	Comments
50	0.3	0.9	0.028	0.0127	0.70	1.02	1.30	0.41	0.05	R & S variance unchanged
50	0.3	0.9	0.014	0.0127	0.73	1.02	1.25	0.38	0.03	R variance reduced 50%
50	0.3	0.9	0.028	0.0064	0.77	1.02	1.28	0.43	0.03	S variance reduced 50%
50	0.3	0.9	0.014	0.0064	0.79	1.02	1.23	0.39	0.01	R & S variance reduced 50%
50	0.3	0.9	0.028	0	0.84	1.02	1.24	0.43	0.01	R variance unchanged, S variance 0
50	0.3	0.9	0	0.0127	0.77	1.02	1.19	0.32	0.01	S variance unchanged, R variance 0
50	0.3	0.9	0.0028	0.0013	0.88	1.02	1.16	0.35	0	R & S variance reduced 90%
50	0.3	0.9	0	0	0.91	1.02	1.14	0.31	0	R & S variance 0

### Examination of demographic parameters

We determined the effects of temporal and geographical (across population) variability. Given 9–10 consecutive surveys, mean recruitment and biological variance were 0.30 and 0.028 (*n* = 11; CV = 0.56), matching results from all these recruitment data. Within a 5 year consecutive period, recruitment = 0.31 with variance = 0.021 (*n* = 24; CV = 0.48). For 3 year consecutive periods, the mean recruitment ratio = 0.29 with variance = 0.018 (*n* = 73; CV = 0.46). Reducing data duration lowered the recruitment variance. Mean values remained consistent.

We examined geographical variation in recruitment data (L:EY) with populations ≥ 20 adult females and ≥ 10 years (*n* = 32), generating a mean L:EY of 0.29. The mean and standard deviation of the variance on these data (i.e., average and standard deviation of the individual variance values) was 0.024 and 0.015, respectively. Hence, there is considerable variability in the variance of L:EY across populations.

Most survivorship data we use originated from desert bighorn sheep within the Peninsular Ranges of California spanning 4–5 years^22^, with CV = 0.14. CVs of survivorships from these populations span 0.10 through 0.19, and CVs for the first 3 years of data average 14% lower than the CV from all years (SD = 0.27). Variation in survivorship also appears geographical and temporal.

The magnitude of change in λ when recruitment shifts from 0.20 to 0.40 and survivorship remains fixed at 0.90 is 0.17. The magnitude of change in λ when survivorship moves from 0.75 to 0.95 and recruitment remains fixed at 0.30 is 0.17. Similar, additive changes in adult female survivorship and recruitment generated comparable results in λ.

### Adult female translocation

**Figure 4:**
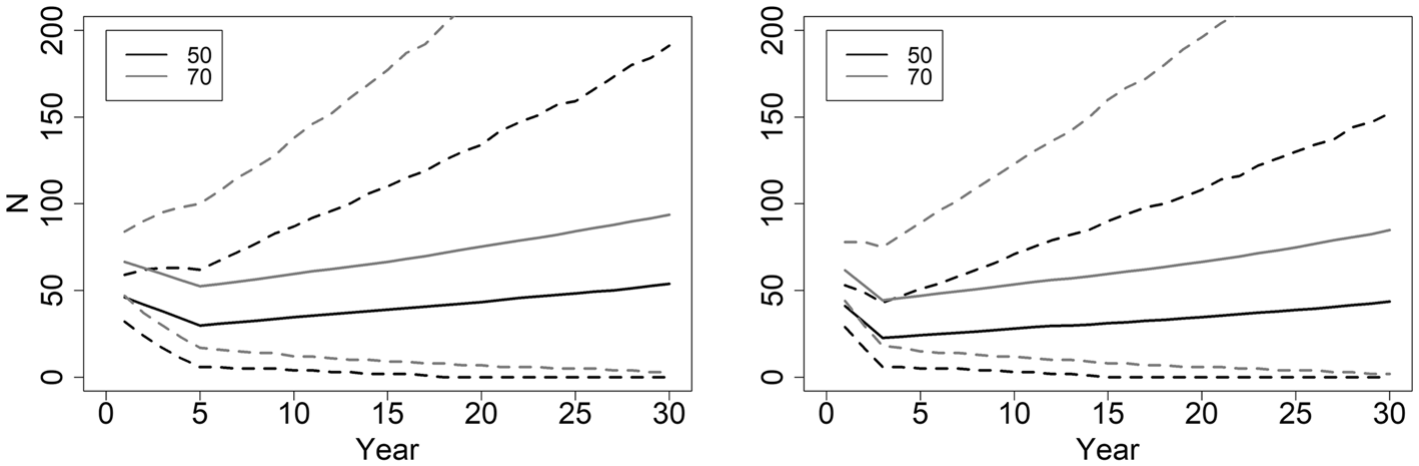
Abundance trajectories of adult females, with removals of 5 adult females during each of the first 5 consecutive years (left), and the removal of 10 adult females during each of the first 3 consecutive years (right). Mean abundances are solid lines with dashed lines representing 90% confidence intervals. Given these 5 or 10 removals, starting abundance must be ≥ 70 adult females for the population to return to a mean of 70 within 30 years without the lower confidence interval reaching 0. Inset values indicate the starting abundances of adult females.

Given a hypothetical removal of 5 adult females during each of 5 successive years (years 1–5), a population of 50 adult females declined to a mean low of 30 (90% CI 6–62) in year 5, and returned to 50 by year 27 (90% CI 0–173). The *QE*(10) is 0.05 and *QE*(30) 0.18, and the lower confidence interval for abundance hits 0 by year 18 (Fig. 4). Populations < 50 did not return to the initial abundance value within 30 years. A population must have a minimum starting abundance of 70 adult females to avoid the 90% lower confidence interval reaching 0. This population attained a low of 52 (90% CI 17–100) in year 5, and returned to 70 by year 17 (90% CI = 8–192; Fig. 4), with *QE*(10) 0.01 and *QE*(30) 0.07.

A population of 50 adult females with 10 removals per year for 3 consecutive years, declined to 23 by year 4 (90% CI 6–47), and returned to 44 by year 30 (90% CI 0–152; Fig. 4). A population requires a starting abundance of 60 adult females to return to 60 by year 28, although the lower confidence interval reached 0 by year 24. Again, a population required a starting abundance of 70 adult females for the lower confidence interval to never attain 0. This hypothetical population reached a low of 46 (90% CI 17–82) in year 4, returned to 70 (90% CI 5–210) by year 22 (Fig. 4), with *QE*(10) 0.01 and *QE*(30) 0.08.

## Discussion

Given *Caprinae* life history and plausible combinations of mean recruitment and adult female survivorship, we evaluated population persistence and estimated population MVP. The values describing adult female survivorship and recruitment, plus the variability we employed match values found in other populations of *Caprinae*. We do not pool data across different *Caprinae* populations or species. Our approach and results directly inform the conservation and management of many *Caprinae*, especially those for which the acquisition of demographic data remains beyond reach.

Our work embodies the characteristics of a high-quality PVA: clear objectives, appropriate demographic data, model structure matching species life histories, stochasticity, examination of extinction probability, appropriate time interval, use of mean values and associated variability^6^. As with most ecological models, the quest for more data remains problematic, not debilitating, and is addressed by creatively and aptly using existing information to generate meaningful results^3^.

Wildlife agencies generate lamb:adult female ratios from *Caprinae* surveys, recognizing that yearlings can be mistaken for adult females, causing miscounts. Excluding yearlings from the ratio’s denominator assumes that no miscounts are occurring, yet an unknown and inconsistent number of yearlings remain in the adult female category across survey events. For these reasons, surveyors of other species, like Dall’s sheep and caribou, pool counts of yearlings and adult females, generating lamb:“adult female-like” ratios instead^15,23–25^.

Managers of *Caprinae* populations can follow these precedents and produce lamb:(adult female + yearling) ratios. Consistency would help standardize methods for building comparisons and meta-analyses across populations of *Caprinae*, while reducing variability across surveys due to differing techniques.

Typically, metrics like elasticity (proportional) and sensitivity (additive) describe the influences of demographic parameters on population growth^13,14,22,26^. For *Caprinae*, when adult female survivorship is 0.90 and recruitment 0.30, the elasticity in survivorship and recruitment are 0.61 (90% CIs 0.40–0.75) and 0.24 (90% CIs 0.13–0.40) respectively (elasticity in young adult survivorship is 0.16 (90% CIs 0.12–0.21). For ungulates in general, the elasticity values for survival tend to be higher than those for recruitment^27^. Our results match this pattern, as the elasticity results indicate that a change in adult survival has a 2.5 times greater effect on λ than an equivalent change in recruitment. Relatedly, other theoretical work reports that demographic parameters with more temporal variability have lower elasticities, indicating less impact on population fitness (e.g.^28,29^).

Our work centers on applications. Since most management actions affect these demographic parameters simultaneously, at issue is the practicality (e.g. feasibility and affordability) of management to increase these parameters, and understanding how such changes could impact λ. For example, imagine a population with mean recruitment of 0.30 and adult survival 0.85, with a biologist interested in increasing recruitment or adult female survival to acquire λ ≥ 1. The answer is to increase either value by 0.02 (Fig. 1, Supplementary Data S1). Similarly, one can set a λ target and determine the amount of recruitment and adult female survival necessary for acquiring it (Fig. 1, Supplementary Data S1).

### Minimum abundance target

A minimum population of 50 adult females meets the persistence criteria, given intermediate levels of recruitment and survival producing λ ~ 1 (Table 2). The risk of population collapse wanes as populations increase above the minimum threshold (Table 2; Fig. 1). For example, a population of ~ 100 adult females always meets persistence criteria (Table 2). Populations of adult females should be somewhat larger than 50 when modest declines (λ ~ 0.97) are suspected, providing a cushion to address the causes of decline, and mitigate further reductions.

Translocation of 5 adult females during each of 5 years, or 10 in each of 3 years, requires a starting abundance of 70 adult females for the population to maintain the persistence criteria, never reach a lower confidence interval of 0, and for the population to return to the starting population size within 30 years. If managers mistakenly target a population having < 50 adult females, the population mean is unlikely to recover to pre-removal levels within 30 years (Fig. 4). The more adult females removed per year will reduce a populations abundance and elevate stochastic effects.

### Applications

These survival and recruitment parameters have high temporal and geographical variability. This uncertainty originates from the variability inherent to survivorship and recruitment data within *Caprinae* populations and demographic stochasticity operating over all age classes, making it difficult to predict exact values of λ and project abundances (Fig. 3). The causes of such variability are complex (e.g. factors such as variation in environmental drivers, predation, management types and timing, the distribution of female reproduction across populations and ages through time), and are rarely monitored. Without data, these factors cannot be explicitly examined. The effects of these factors, however, are contained in the empirically-derived estimates for survival and recruitment, and their associated variability.

We examined the effects of time and management actions on reducing variation in recruitment and adult female survivorship. We began by halving the variance in recruitment, given its empirical basis. For all recruitment data, when the temporal window is short (3 years), recruitment variability declined to nearly half the overall mean variability. Also, the NMDGF operate a fenced facility (6.2 km^2^) for desert bighorn sheep, to translocate surplus animals elsewhere. These sheep remain wild, yet receive year-round water and rare predation events. This population has biological variance in recruitment approximately half that of the variance of other populations of desert bighorn in the southwest U.S. (variance = 0.015). The mean recruitment value (L:EY) recorded within this facility (0.56; N = 18 years; CV = 0.22) is nearly double the amount of recruitment calculated from the other populations of desert bighorn sheep.

For survival, most of these data represented desert bighorn sheep populations whose abundance were declining (disease, predation, habitat loss^22^). Survivorship of adult females in endangered or declining populations of ungulates could be lower or more variable than in demographically viable populations^30–32^.

Management actions can reduce variation in recruitment and survival. However, the simulations halving variance in recruitment or survival received minimal increases in precision in λ or abundance (Table 4; Fig. 3). Halving variability in adult female survivorship and recruitment, or eliminating it, moved the probability of λ < 1 to 0.39 and 0.31 (from 0.41) while reducing *QE*(30) from 0.05 to 0.01 and 0 respectively (Table 4). Therefore, in application, halving variance in recruitment (i.e., calculated from predator-free populations) and survival (i.e., from predator control) does not generate much change in population trajectories.

Management actions can also change the mean values of demographic parameters. For instance, populations of grazing mammals lacking predation have higher growth rates with less variability^33^. Therefore, when populations experience predation, predator control can improve population sizes of *Caprinae* by raising the survival of adult females and recruitment^12,34,35^. Survival of adult females can increase 5–12% with predator control, and survival with predation is often twice as variable as populations without predation^12^. Imagine predator control boosted mean adult female survival from 0.90 to 0.95^12^ and recruitment from 0.30 to 0.50 (similar to the NM penned facility), while reducing the survival and recruitment variance by half (^12^; NM penned facility). Population growth would increase 20%, from λ = 1.02 to 1.22. Mean abundance would climb from 56 adult females to 135 by year 5.

Predators are important components of ecosystem health^36,37^, so predator management requires conscientious approaches. Predator control (or perhaps reduction in conspecific, alternative prey) could be applied to improve *Caprinae* populations with < 50 adult females. Larger populations should withstand background levels of predation, when stable or growing. Curbing declines in larger populations, or attaining targets in population growth or abundances could also warrant predator control, to accomplish management objectives. Monitoring the abundance and recruitment of *Caprinae* populations helps identify conditions suggesting management actions like predator control, while indicating when the desired project objectives are achieved (i.e.* Caprinae* population responses). Factors like environment, climate and disease influence population abundance too, and are worth considering during such monitoring^38^.

Provision of supplemental water can increase the distributions of *Caprinae*^39,40^. Supplemental water, however, could increase predator distributions (directly or indirectly), potentially aggravating predation issues affecting adult female survival and recruitment^41,42^. Other hypotheses, like temporal reductions in water supplementation, may also reduce predator presence within *Caprinae* ranges^43,44^. Alternative management tools for improving *Caprinae* abundances are supplemental feeding and disease control, challenging and costly to pursue at large scales.

The variability in adult female survival and recruitment means that 1 year’s recruitment or survival cannot accurately predict the next, and historical trends in population abundance may have little bearing on future performance (Fig. 3). Revealing management contributions to population growth is also challenged by the variability inherent within the demographics of *Caprinae* populations. This situation makes *Caprinae* management reactive, with management decisions based on the short-term monitoring results for a population. The repeated acquisition of accurate abundance data, however, builds a more proactive paradigm.

### Monitoring *Caprinae*

Population vital rates and their importance to population growth often varies across populations and within a population over time (our results^31,32,45,46^). Indeed, within and across populations of *Caprinae,* the annual differences in survival and recruitment are substantial^47,48^. Some temporal and geographical variation is biological, and some stems from the use of different methodologies for quantifying variability. Hence, analytical projections of future abundance based on survival and recruitment data from one population are unlikely to apply to other populations for the same or different species. Understanding a given populations status requires directly monitoring that population.

Frequent monitoring of many populations requires simple and affordable methods for identifying a population’s status (abundance, growth). We discovered the threshold of 50 adult females with three types of demographic parameters: abundance, survival and recruitment. Monitoring the threshold is achievable with two of them.

Adult female survival is the most challenging and costly parameter to acquire, unattainable for most biologists managing *Caprinae* populations. Indeed, we struggled to locate data describing adult female survival given a species extensively studied. Future monitoring and analyses, therefore, do not require survival data, but can rely solely on measures of abundance and recruitment.

Minimum counts are the primary method for monitoring *Caprinae* species. This method is appropriate if precision measurements are not required and acknowledgement that data are biased low. With minimum counts, management actions dependent upon abundance triggers (i.e., 50 adult females) would be identified early, providing a conservative approach to population management. In the short term, populations can be prioritized for conservation using minimum abundances, but these data make it difficult to generate longer-term conclusions about population sustainability.

When minimum counts or mean abundance estimates for adult females are well above 50 (i.e. ~  ≥ 75), the population is likely to persist (unless conditions change). When populations occur at or near the threshold value, recruitment estimates associated with these abundance estimates (and trends) help biologists infer if the population is stable or growing, and if the minimum abundance threshold were reached. For example, imagine a population with known abundance of 70 adult females. A survey was conducted, producing an estimate of 65 adult females (90% CI 44–86 (20% CV)), with a recruitment estimate (Lamb:(adult female + yearling)) > 0.30. Trends in abundance from prior surveys indicate an increase. A reasonable interpretation of these data is that the population is likely at or above the threshold and growing.

We used abundance trends because of the high variability in recruitment trends (demonstrated herein). If either abundance, abundance trend or recruitment shows a concerning decline, it should initiate work to discern the cause, as the population may no longer be sustainable.

While the acquisition of accurate abundance data can be challenging^48–50^, researchers have posed tenable solutions for meeting these challenges^50,51^. These methods rely on methodological changes in aerial surveys, or replacing aerial surveys with motion activated cameras, that collaterally identify predators and their relative abundances.

For annually-reproducing *Caprinae* that begin reproduction in their second to third year, the minimum abundance threshold of 50 adult females should apply. Source populations for translocation have a 70 adult female abundance threshold, assuming translocations of 5 or 10 adult females per year, in each of 5 and 3 years respectively. For *Caprinae* with different life histories, our model code is easily modified to accommodate those changes.

Hence, our work extends beyond *Caprinae*. The population projection matrix model we developed can be applied to any species reproducing on an annual cycle. Users specify parameters such as the number of stage-classes, mean and variance of recruitment and adult survival, starting population size, correlation between recruitment and survival, sex-ratio at recruitment, and number of translocations (Supplementary Data S1). As exemplified herein, results will identify a population's’minimum threshold for persistence, which subsequently informs threat assessments, harvest quotas and the triage of conservation activities aimed at recovering ailing populations.

## Methods

**Figure 5:**
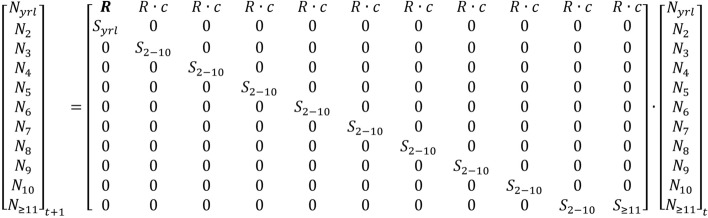
Population projection matrix used for simulating *Caprinae* population growth and the abundance of adult females. The first recruitment entry (bold R) is a necessary computational step to correctly represent reproduction, and account for the observer misclassifications of yearling male and females as adult females. Symbols indicate the following: *R* = recruitment, *S* = adult female survivorship, *c* = 0.5 (sex ratio), *N* = Age class.

Our matrix model simulated population trajectories given various combinations of starting abundances, recruitment, adult female survival values, and translocation scenarios (Fig. 5). We predicted population growth, adult female abundance and determined extinction and quasi-extinction (*QE)* probabilities through time. We defined extinction when abundance = 0 adult females, and *QE* when abundance ≤ 5 adult females. Results from these simulations enabled us to identify a minimum target abundance of *Caprinae* to ensure population persistence^4^. We defined persistence as a population with ≤ 0.01 chance of *QE* in 10 years and ≤ 0.10 chance of *QE* in 30 years.

### Demographic parameters

We examined peer-reviewed publications by searching “Google Scholar” for mean recruitment and survival parameters reported for *Caprinae* species, to ensure that model parameters correspond with data representing a diversity of *Caprinae*. Our searches covered all years, and included ≥ 1of the following keywords: Abundance, Ammotragus, argali, Capra, Capricornus, Caprinae, Caprini, chamois, goat, goral, ibex, mouflon, Naemorhedus, Oreamnos, Ovibovini, Ovis, recruitment, Rupicapra, Rupicraprini, serow, sheep, survival, survivorship, takin, tur, urial. The simulations and analyses also required an accurate measure of biological variance in *Caprinae* recruitment and adult female survival to ensure that robust measures of variability accompanied the resulting predictions. Recruitment and survivorship surveys often differ in their methodology (e.g. aerial, ground counts, telemetry), timing and duration. To ensure a robust understanding of the variability in recruitment and survivorship data entering the simulations, we avoided arbitrarily selecting values from the literature. Instead, we calculated the variability in these parameters from data representing desert bighorn sheep. We compared the variability values gained from our calculations to those presented for other *Caprinae* to ensure relevance and transferability in results.

We found few publications describing ewe survival for *Caprinae*. Therefore, to compute variability, we relied on survival data for desert bighorn sheep from three publications with studies spanning multiple, sequential years^22,52,53^. To calculate variability in recruitment, we acquired recruitment data on desert bighorn sheep from three state agencies: Arizona Game and Fish Department (AZGFD; 1958–2008; 90 locations), California Department of Fish and Wildlife (CDFW; 1991–2010; 13 locations), and New Mexico Department of Game and Fish (NMDGF; 1979–2016; 6 locations). Most *Caprinae* bear one young per year (periodic twinning can occur; an outcome more common in some species), with females reproducing during their second or third year until death^1^. Desert bighorn sheep share these characteristics. We estimated the biological variance (i.e., the component of variance due to temporal and environmental variation) of survival and recruitment by using the variance discounting procedures which removes the sampling variation^54^.

### Population dynamics

The stage-structured population projection matrix model used 11 age-based categories (Fig. 5). Our models used age-class delineations corresponding to a post-breeding census. We considered lambs (or kids) < 0.5 years, yearlings as ages ≥ 0.5 and < 1.5 years, and adult females as ≥ 1.5 and ≤ 12.5 years (herein, the term “lamb” is also interchangeable for “kid”, when the sentence is used in context for *Caprinae* species). We set yearling survivorship as 10% lower than adult female survivorship, a reduction based on prior work with bighorn sheep^55^. We considered prime aged animals ages 2–10 years, with survival equivalent across these age classes^55,56^. Older females (≥ 11.5) received a 0.43 proportional reduction in survival from adult female survival (calculated from life history table^56^). We set the amount of variation in yearling and older female survival to equal the variation in adult survival.

We integrated demographic stochasticity (inherent randomness that arises from the discrete nature of individual animals^57^) and biological variation into the simulation. Biological variation was derived from the empirical recruitment and survival data. For each year and iteration, we randomly selected adult female survival and lamb recruitment using the incomplete beta function, which allows for correlated vital rates^58^. We assumed the vital rates were correlated at 0.40 (calculated from^22^). We used the first and second moments to calculate the scale and shape parameters of the beta distribution^58,59^. We assumed a 50 male:50 female sex ratio of recruited lambs, normally distributed with a variance of 0.01^60^. We incorporated demographic stochasticity as a binomial random variable through the random generation of survivors and recruits. The number of trials (*n*) matched the number of individual adult females in a given stage, with the parameter *p* of the binomial distribution a random variable simulated from the beta distribution.

Our simulations used combinations of mean survival and recruitment values to estimate population growth (λ). We allowed mean survival to range from 0.70 to 0.98, mean recruitment from 0.05 to 0.90 (to account for scenarios with and without twinning) and starting abundance from 10 to 250 adult females. Biological variance remained fixed to empirical values. We explored the effects of biological variation on population dynamics in subsequent simulations by artificially halving and eliminating the recruitment and survivorship variances. We also identified how changes in survivorship and recruitment affected λ.

While multiple factors affect the mortality of adult females (e.g. predation, starvation, disease), declines in abundance also occurs when adult females are translocated from a source population to another population. Translocations of adult females should be an acceptable practice, provided the source population does not encumber a high risk of *QE* resulting from removals. When desert bighorn sheep are translocated from a population, it is common for managers to pursue sequential years of removals. Therefore, we examined the effects of adult female removals on population stability by examining two translocation scenarios: removing 5 adult females consecutively for 5 years (25 total removals) and removing 10 adult females over 3 consecutive years (30 total removals). These translocation simulations fixed recruitment and survival parameters to generate a stochastic λ = 1.02 (recruitment = 0.30, survivorship = 0.90, with biological variability), as populations with λ ≤ 1.00 would not recover from the translocation event.

Simulations estimated abundance and λ for each combination of survival and recruitment, every year, for 30 years, with 10,000 individual iterations. We tested model operation to ensure results replicated the values of mean recruitment, mean survival and their respective variability. For each simulation we determined extinction and *QE* each year.

Data describing lamb:adult female ratios typically represent recruitment, a combination of adult female fecundity and lamb survival. Lambs are born in spring and surviving lambs are counted and considered recruited during fall surveys (~ 6 months post-birthing). Our model relies on recruitment ratios, as these data are routinely collected by *Caprinae* biologists. For *Caprinae*, age-specific recruitment data are rarely (if ever) recorded during population monitoring. The variability in the lamb:ewe data accounts for any differences in reproduction across age classes through time and across populations.

Management agencies report lamb:adult female ratios differently, with some agencies including or excluding yearlings in the ratio denominator. We examined these lamb:adult female data, finding that yearlings (of both sexes) were often misclassified as adult females. This outcome is not unusual, as surveys for other species often misclassify yearling male and females as adult females^25^. Grouping yearlings and adult females into a ratio of lamb:‘adult female-like’ is also a common solution, for representing recruitment in *Caprinae* and other ungulates^15,24,61^. Therefore, we employed lamb:‘adult female-like’ ratios in the simulations.

Mathematically, we adjusted the population projection matrix to account for the low bias in recruitment, occurring from the inclusion of yearling males and females in the denominator of the ratio. This simple adjustment multiplied the recruitment ratio by 2 in the first column of the matrix (bold R representing the first age category; Fig. 5; Supplementary Data S1). To explain, imagine a hypothetical population consisting of 80 adult females (E) producing 30 lambs (L), with 10 male and 10 female yearlings (YM and YF respectively). The conventional lamb:adult female ratio (L:E) is 0.375. An unknown number of yearlings, however, are misclassified as adult females. When all yearlings are included in the denominator of the lamb:adult female ratio (L:(E + YM + YF)) the recruitment ratio is biased low (0.30). The traditional matrix would only include R in the 2nd–11th column of the matrix (the bold R omitted in the matrix within Fig. 5), which would produce 24 lambs (80 × 0.30 = 24). Yet we know from the example, that 30 lambs were produced. Alternatively, if R occurred in all columns of the matrix, the result is 27 lambs (80 × 0.30 + 10 × 0.30 = 27) and lamb production remains biased low. This problem arises because male and female yearlings are not reliably distinguished. To correct this problem, we multiplied R by 2 in the first column (80 × 0.30 + 10 × 2 × 0.30 = 30), which accounts for yearling males. The 30 lambs includes both sexes, and since our simulation represents adult females, R is multiplied by the lamb sex ratio (c = 0.5).

## Supplementary Information

Supplementary Information 1.

Supplementary Information 2.

Supplementary Legends.

## Data Availability

Program and sample data to run analyses are included.
