# Supplementary material for: The abundance and persistence of Caprinae populations
Source: Sci Rep. 2022 Aug 15;12:13807. doi: 10.1038/s41598-022-17963-w (PMC9378773; doi:10.1038/s41598-022-17963-w)
Supplement: Supplementary file 3 — Supplementary Legends. [file 41598_2022_17963_MOESM3_ESM.docx]

Supplementary Data S1: A text file with R code for estimating a population’s minimum threshold for persistence. Users can modify many parameters, such as: starting adult female abundance, adult female survivorship, recruitment, variability in survivorship and recruitment to predict future abundance, growth rate (λ), and population trend. Code is attributed for ease of use.

Supplementary Data S2: A .csv file containing example input for running Supplementary Data S1 R code
